# A new setup for single surgeon paediatric supracondylar fracture pinning

**DOI:** 10.1308/rcsann.2024.0018

**Published:** 2024-11-01

**Authors:** K Dogramatzis, M Imam, A Cameron-Smith

**Affiliations:** Ashford and Saint Peter’s Hospitals NHS Trust, UK

## BACKGROUND

Manipulation and pinning of displaced paediatric supracondylar fractures is an operation frequently performed out of hours due to impaired perfusion or threat to the overlying skin.^1^ We describe a single-surgeon theatre setup that allows for reduction, easy access for pinning and avoidance of intraoperative elbow manoeuvres, while also being easy to perform without an assistant.

## TECHNIQUE

**Figure 1:**
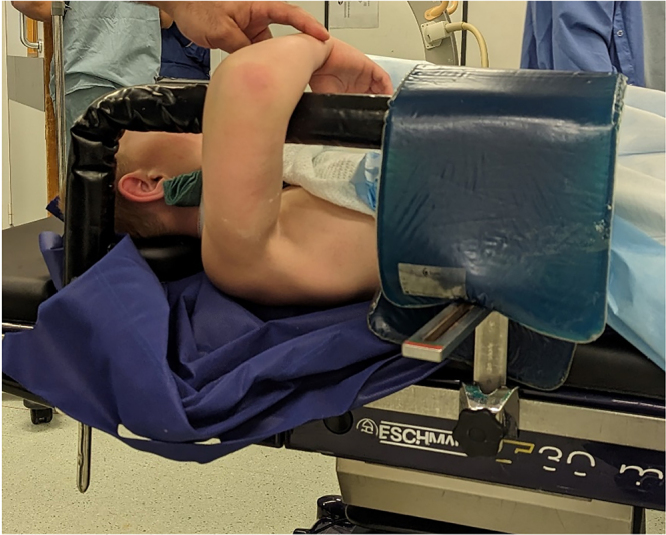
Theatre setup before prepping/draping. A lateral support lies under the gel pad to keep the L bar from rotating.

**Figure 2:**
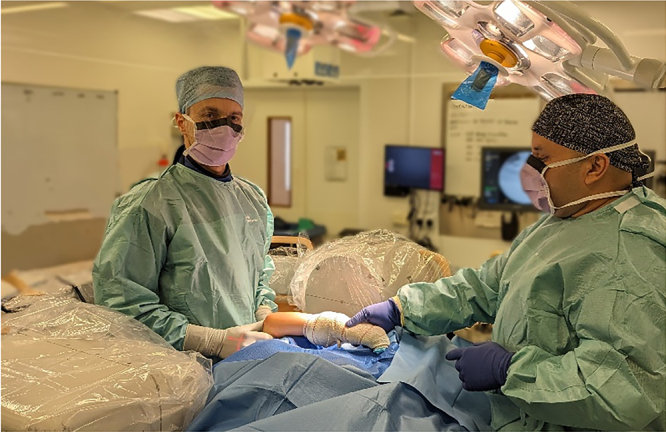
Intraoperative positioning. The scrub nurse seats to the right of the surgeon, outside the C-arm. Note the ergonomic position and ease of access for K-wire insertion.

**Figure 3:**
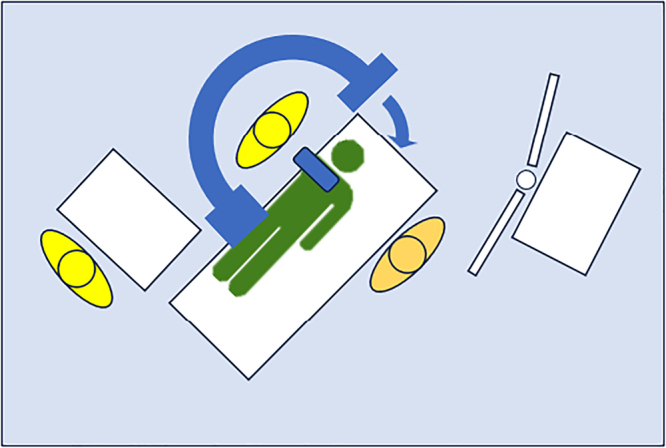
Schematic representation of theatre setup. Arrow points towards the direction of rotation of the intensifier for AP views.

The patient is positioned supine with the affected side close to the edge of the operating table. The arm is lifted and a padded horizontal bar support is positioned 2–3cm proximal to the elbow crease, so that the hand of the patient can rest on his/her chest with elbow flexion (Figure 1). The limb is prepared and draped from the proximal humerus distally. The surgeon stands over the elbow and the image intensifier is positioned around his/her waist (Figure 2). Inline traction and reduction manoeuvres are performed by the surgeon and the reduction is maintained by the assistant/nurse applying traction to the patient's hand from the other side of the operating table (Figure 3). Anterior posterior radiographs are obtained by partially extending the elbow and the intensifier rotating around the elbow.

### Advantages

•  Ease of access for reduction
•  Good quality lateral elbow imaging
•  Comfortable surgeon position for K-wire insertion
•  Minimal risk of fracture displacement as the limb does not need to be moved for x-rays or K-wire access.
•  No need for an assistant

## DISCUSSION

We found this setup to be effective at lowering operation times, avoiding loss of reduction and K-wire drape perforations, while also allowing mini-open reduction and ulnar nerve exploration if needed. Due to the x-ray beam orientation, the surgeon's radiology apron needs to provide adequate side coverage. We would advise a 0.5mmPb or thicker circumferential apron.
